# Why women bleed and how they are saved: a cross-sectional study of caesarean section near-miss morbidity

**DOI:** 10.1186/s12884-016-1182-7

**Published:** 2017-01-09

**Authors:** S. Maswime, E. J. Buchmann

**Affiliations:** 1 Wits Obstetrics and Gynaecology Clinical Research Division, Johanesburg, South Africa; 2 Department of Obstetrics and Gynaecology, University of the Witwatersrand, Johannesburg, South Africa

**Keywords:** Near-miss, Caesarean section related haemorrhage, Bleeding during and after caesarean section

## Abstract

**Background:**

Maternal deaths from ‘bleeding during and after caesarean section’ (BDACS) have increased in South Africa, and have now become the largest sub-cause of deaths from obstetric haemorrhage. The aim of this study was to describe risk factors and causes of near-miss related to BDACS and interventions used to arrest haemorrhage and treat its effects.

**Methods:**

Cross-sectional prospective study in 13 urban public hospitals in South Africa, from July to December 2014.

**Results:**

There were 93 cases of near-miss related and 7 maternal deaths related to BDACS. The near-miss rate was 2.1/1000 live births, and the case fatality rate was 3.5/10 000 caesarean sections. Associated near-miss risk factors were previous caesarean section in 60% of multiparas, pre-operative anaemia (55%), abruptio placentae (20%) and placenta praevia and/or accreta (20%). Atonic uterus (43%) was the most frequent anatomical cause of bleeding for near-miss, followed by surgical trauma (29%). The median duration of the operations resulting in near-miss was 90 min, with 81% noted as difficult by the surgeon. Interventions in cases of near-miss included second-look laparotomy (46%), hysterectomy (41%), B-Lynch brace suture (9%), intensive care unit admission (32%) and red cell transfusion ≥3 units (21%).

**Conclusion:**

Cases from maternal near-miss from BDACS were frequently associated with pre-operative risk factors. Extensive life-saving interventions were required during and after the operations. An important factor in initiating the sequence of interventions is the realisation by the surgeon that the caesarean section is difficult, so that the progression from uneventful operation to near-miss to death can be arrested.

## Background

The safety of caesarean sections has recently come into question in South Africa. Maternal death resulting from ‘bleeding during and after caesarean section’ (BDACS) is becoming increasingly frequent. The triennial reports of the government’s confidential enquiries into maternal deaths recorded 78 such deaths in 2002–2004, 140 in 2005–2007, 180 in 2008–2010, and 221 in 2011–2013 [1–4]. Maternal death from BDACS is now the largest sub-cause of death from obstetric haemorrhage in South Africa [4]. The increase in maternal deaths has been attributed to a rising national caesarean section rate, but this increase exceeded the rise in caesarean section rate, which increased from 12.7% in 2001/02 to 20.8% in 2012/13 [5]. Other possible reasons, for which data are lacking, related to changes in health care quality, such as a lower standard of surgical skill with increased risk of bleeding [6], failure to detect antepartum risk factors, and possibly substandard labour management, anaesthesia and post-operative care.

Globally, there are insufficient data on maternal deaths related to BDACS, especially in terms of clinical and surgical detail. Even with the recent increase in deaths, these events are so rare that meaningful prospective studies are difficult to do. A helpful alternative is to study ‘near-miss’ related to BDACS. A maternal near-miss is a woman who survives severe complications during pregnancy, childbirth or postpartum. The study of near-miss is an increasingly useful tool in obstetric clinical audit, because of the larger number of cases available for study compared with maternal deaths, and because valuable information can be obtained on how clinical and surgical and clinical management obstacles are overcome after the onset of an acute complication [7].

To our knowledge, there has been no published scientific work focusing on maternal near-miss related to BDACS. Studies or audits on near-miss could improve understanding of the evolution of severe bleeding at caesarean section, and describe opportunities taken and missed to control the bleeding and treat its consequences. The information gained may inform quality improvement and interventions to reduce near-miss and death from BDACS.

The aim of this study was to determine the risk factors and causes of near-miss related to BDACS and to describe the clinical and surgical interventions undertaken.

## Methods

This was a cross-sectional prospective study in 13 government hospitals in southern Gauteng province, South Africa, from July to December 2014. Ethical approval was obtained to conduct this study from the University of the Witwatersrand’s Human Research Ethics Committee. Informed written consent was obtained from the participants.

The city of Johannesburg is the geographic and economic centre of southern Gauteng province. There are 18 government hospitals with maternity services in southern Gauteng, with about 100 000 births per annum. Three of these hospitals are university-attached tertiary referral hospitals. The 13 hospitals in the study included the three tertiary hospitals, as well as seven regional referral hospitals, and three district hospitals. The district hospitals are staffed by non-specialist doctors and have relatively small case loads. The regional hospitals all have at least one specialist, as well as medical officers who are responsible for most of the daily clinical activities. The tertiary hospitals are better resourced in terms of equipment and facilities, and include university faculty staff and residents training to become specialists.

The World Health Organization (WHO) Intervention criteria were used to identify cases of near-miss, and were modified to suit this population [7]. Only near-misses related to BDACS were included in this study. A near-miss related to BDACS was any woman with a gestational age ≥24 weeks, or who delivered a baby weighing ≥500 g, who had a caesarean section with a combined intraoperative and postoperative blood loss ≥1000 mL, and at least one of the following: red cell blood transfusion ≥3 units, emergency hysterectomy, repeat laparotomy, transfer to a higher level of care, started or restarted postoperative ventilation, use of inotropic drugs, acute dialysis, cardiopulmonary resuscitation and admission to an intensive care unit. Women with ruptured uterus, extra-uterine pregnancy, and where another cause of haemorrhage was the primary reason for bleeding, were excluded.

The researcher trained obstetric staff in each hospital on how to identify a near-miss from BDACS. Each time such a case was identified, a staff member notified the researcher telephonically. The researcher then travelled to the hospital and screened the file for study eligibility. Data were collected from maternal case records, and supplemented, where necessary, from patients themselves or from staff. Informed consent was obtained from the patients. Data collected from the clinical notes were antenatal history, details about the previous pregnancy and blood test results, reason for admission, clinical management, and events leading to the decision for caesarean section. Details about the caesarean section, the findings and the complications were recorded, as well as postnatal observations and interventions. The researcher made regular visits to all hospitals to ensure ongoing surveillance. Numbers of maternal deaths related to BDACS were also recorded.

A sample of 100 near-misses related to BDACS was envisaged to provide reasonable confidence intervals for observed percentage frequencies. With a sample of 100 subjects, an observed percentage has 95% confidence limits that are not more than 10% above and below it. Data analysis employed quantitative techniques, using Stata version 11 software (Statacorp, College Station, TX, USA). Descriptive data were analysed using medians, ranges and interquartile ranges for continuous variables, and proportions with percentages for categorical variables. Comparisons of categorical variable frequencies were made using Fisher’s exact test. A *p*-value <0.05 was used to determine statistical significance.

## Results

The hospitals reported 93 near-misses related to BDACS, as well as 7 maternal deaths. The total number of deliveries at all the hospitals was 46 775, of which 20527 were caesarean sections (43%). The near-miss related to BDACS rate was therefore 2.1 per 1000 (93/43474) live births, and the BDACS- related case fatality rate was 3.4 per 10 000 caesarean sections.

Obstetric data and risk factors for near-misses are shown in Table 1. Teen pregnancy (*n* = 2) and parity ≥4 (*n* = 5) were infrequent. Twenty women were nulliparous and 73 were multiparous. Forty-four (60.3%) of the multiparous women had previous caesarean sections. Anaemia prior to caesarean section (haemoglobin level <11.0 g/dL) was found in 49 of 89 women tested (55.1%). Thirty-six women (40.4%) were HIV infected. Clinical risk factors included hypertensive disorders in pregnancy in 26 (28.0%), abruptio placentae in 19 (20.4%), and placenta praevia and/or accreta in 19 women (20.4%) Nine of the 19 women with abruptio placentae had a hypertensive disorder. The main primary indications for caesarean section were fetal distress (*n* = 14; 15.1%), abruptio placentae (*n* = 13; 14.0%) and placenta praevia (*n* = 12; 12.9%). Eleven women had caesarean section for prolonged labour, 7 in the first stage of labour (7.5%) and 4 in the second stage (4.3%). Twenty-four infants were stillborn, 17 of them in association with abruptio placentae. The surgeons who started the caesarean sections were non-specialists in 29 cases (31.2%), residents in 54 cases (58.1%) and specialists in 7 cases (7.5%). Seven caesarean sections (7.5%) were done in district hospitals, 22 (23.7%) in regional hospitals and 64 (68.9%) in tertiary hospitals.

**Table 1:** Obstetric characteristics and risk factors for maternal near-miss from bleeding during or after caesarean section (*n* = 93, unless otherwise specified because of missing data)

	Number	Percent
Maternal age (years):		
< 20	2	2.2
20–34	69	74.2
≥ 35	22	23.7
Parity:		
0	20	21.5
1–3	68	73.1
4	5	5.4
Attended antenatal clinic	83	89.2
Previous caesarean section	44	47.3
Haemoglobin level prior to caesarean section (g/dL) (*n* = 89):		
< 8.0	11	12.4
8.0–10.9	38	42.7
≥ 11.0	40	43.0
HIV infection (*n* = 89)	36	40.4
Antenatal weight ≥90 kg (*n* = 79)	8	10.1
Caesarean section (*n* = 91):		
Elective (planned)	11	12.1
Emergency not in labour	28	30.8
Emergency in first stage	44	48.4
Emergency in second stage	8	8.8
Hypertensive disorder of pregnancy	26	28.0
Abruptio placentae	19	20.4
Placenta praevia/accreta:		
Placenta praevia	7	7.5
Placenta accreta	9	9.7
Placenta praevia and accreta	3	3.2
Birth weight (g) (*n* = 85):		
< 1000	6	7.1
1000–2499	33	38.8
≥ 2500	46	54.1

The most frequent cause (anatomical site) of haemorrhage was atonic uterus (*n* = 40; 43.0%), followed by uterine incisional bleeding (*n* = 27; 29.0%), placental site bleeding (*n* = 16; 17.2%), bleeding at the bladder reflection (*n* = 8; 8.6%), pampiniform venous plexus bleeding (*n* = 5; 5.4%) and abdominal wall bleeding (*n* = 4; 4.3%). Two or more causes of bleeding occurred in some women. In 14 women (15.1%), the cause of haemorrhage was not specified. The causes of bleeding were compared between women with previous caesarean section, placenta praevia/accreta, abruptio placentae, and the remainder (considered as having no surgical risk). There were significant associations between placenta praevia/accreta and placental site bleeding (*p* < 0.01), and between abruptio placentae and atonic uterus (*p* = 0.04). There was a trend to more frequent pampiniform plexus and abdominal wall bleeding in women with previous caesarean section (Table 2).

**Table 2:** Caesarean section near-miss haemorrhage anatomical sites, according to presence or absence of specific prior surgical risk factors

	Previous caesarean section only (*n* = 23)	Placenta praevia/accreta (*n* = 19) – includes 13 previous caesarean sections	Abruptio placentae (*n* = 19) – includes 8 previous caesarean sections	No surgical risks anticipated (*n* = 32)
Uterine incision	5	5	3	14
Atonic uterus	9	4	14*	13
Placental site	1	14*	0	1
Bladder reflection	1	3	2	2
Pampiniform plexus	3	0	1	1
Abdominal wall	3	1	0	0
Not specified	3	1	2	8

*Comparison for placental site bleeding, placenta praevia/accreta v. no surgical risks: *p* < 0.01

*Comparison for atonic uterus, abruptio placentae v. no surgical risks: *p* = 0.04

The surgeons noted difficulty in 76 operations (81.7%). The duration of surgery was estimable in 80 cases, and was <60 min in 14 (17.5%), 60–119 min in 34 (42.5%) and ≥120 min in 32 (40.0%). The median duration was 90 min, with a range of 23–345 min. Forty-nine women (52.7%) received blood transfusions during their caesarean sections. Medications and procedures used to arrest bleeding (in addition to routine intravenous oxytocin) included ergometrine, misoprostol, prostaglandin F2-alpha, tranexamic acid, B-Lynch brace suture, systematic uterine vessel ligation (devascularisation), and intrauterine balloons (Table 3). The median blood loss at caesarean section (estimated by the surgeons in 69 cases) was 1000 mL, with a range of 250–3600 mL. Second-look emergency laparotomy was performed in 43 women, of whom 18 underwent hysterectomy (total hysterectomies: *n* = 38; 40.9%) and 8 had B-Lynch compression sutures (of which 4 failed, requiring hysterectomy). Fourteen women had only resuturing or uterine vessel ligation done, and 5 required no specific haemostatic procedure. Seventy-four women (79.6%) received three or more units of red cell transfusion during their hospital stay. The median lowest postoperative haemoglobin level was 6.05 g/dL (*n* = 90), with a range of 2.8–12.5 g/dL. Thirty-six of 78 women in whom the platelet count was measured had a postoperative thrombocytopenia <100 000/μL. Seven women had severe acute kidney injury (serum creatinine ≥250 μmol/L), and 6 required dialysis. Thirty women (32.3%) were admitted to intensive care units postoperatively. Eleven women (11.8%) received inotropic drugs. Ten women (10.8%) were transferred by ambulance to higher levels of care.

**Table 3:** Intraoperative interventions during caesarean section in cases of near-miss haemorrhage (*n* = 93 unless otherwise specified because of missing data). Multiple interventions were used in some cases

	Number	Percent
Red cell blood transfusion (*n* = 89)		
None	40	44.9
1 unit	8	9.0
2 unit	22	24.7
≥ 3 units	19	21.3
Intramuscular or intravenous ergometrine	19	20.4
Oral or rectal misoprostol	8	8.6
Intramyometrial prostaglandin-F2-alpha	10	10.8
Intravenous tranexamic acid	6	6.5
Systematic uterine vessel ligation (devascularisation)	7	7.5
Intrauterine balloon^a^	4	4.3
B-Lynch compression suture^a^	15	16.1
Hysterectomy	20	21.5

^a^Three of the intrauterine balloons failed to stop the bleeding, and two of the B-Lynch compression sutures (in whom balloons were not used) failed. All five failures went on to hysterectomy

The seven maternal deaths included a case of placenta accreta with previous caesarean section, who required a hysterectomy, and one woman with placental abruption who died four days postoperatively from multi-organ dysfunction. The remaining five women had no surgical risk factors, and all had intraoperative blood loss ≤700 mL.

## Discussion

This study, from a middle-income urban setting, found that about one in 216 caesarean sections are complicated by severe morbidity from bleeding, and that about 1 in 14 women with severe morbidity progress to maternal death. The near-miss definition seemed appropriate as applied in this study, providing a sample of women severely affected by bleeding, evidenced by a very low median postoperative haemoglobin level of 6.05 g/dL.

The most important common risk factors for near-miss related to BDACS were preoperative anaemia (over half had haemoglobin <11.0 g/dL), and previous caesarean section (in over half of multiparas). A rising caesarean section rate increases not only the number of caesarean sections currently, but adds women with previous caesarean section to those who will become pregnant in future. This is one of the reasons for calls to prevent the first caesarean section in women [8]. The HIV infection rate, while high, is not statistically significantly higher than the 28.7% prevalence in pregnant women in this part of South Africa (*p* = 0.05) [9]. Anaemia limits the body’s response to blood loss, and makes any caesarean section potentially hazardous. Previous caesarean section is well known as a cause of overall surgical difficulty [10]. Accordingly, multiple causes of bleeding were found in women with previous caesarean sections, including extrauterine causes such as pampiniform plexus and abdominal wall injury. The other two important risk factor categories, abruptio placentae and placenta previa/accreta, are uncommon conditions, yet occurred in large numbers in this series. The published literature supports our finding of uterine atony being the most common cause of post-partum haemorrhage, and placental site bleeding associated with placenta praevia/accreta [11]. Previous caesarean section itself is also associated with placenta praevia/accreta [12]. A number of women had no specific surgical risk factors, and bled severely from a variety of sources. The absence of risk factors gives no guarantee of an uncomplicated caesarean section and postoperative course.

In 76% of near-misses related to BDACS, the caesarean sections were noted by the surgeons as difficult, and early interventions were made intra-operatively. A range of life-saving options was used, usually in combination, to manage the source and effects of bleeding. Medical management included utero-tonics, tranexamic acid, inotropic support and blood transfusion, with recourse to emergency transport if needed, and intensive care, suggesting the elements of a well-resourced functional health service. Surgical options included frequent use of uterine vessel ligation, B-Lynch compression sutures and hysterectomy, both at caesarean section and at second-look emergency laparotomy, suggesting the presence of skilled obstetric surgeons. The few attempts at intrauterine balloon tamponade were mostly unsuccessful, but this may reflect the characteristics of this group of women. Balloon tamponade works best when placed early in severe postpartum haemorrhage after vaginal birth [13].

This study has certain strengths and limitations. Strengths were a robust near-miss definition and a sample size that allowed description of prevalence and relationships. Another strength is the clinical detail obtained from inspection of all women’s clinical notes by a specialist obstetrician. The reliance on record review for clinical information is also a limitation, with some data missing. Limitations include the possibility that some near-miss cases were not included, because the researcher and the trained contacts at the hospitals could not be in attendance at all births. However, it is certain that all maternal deaths from BDACS were included, because these are subject to compulsory confidential enquiries.

Further research should focus on detail in risk factors, surgical findings and interventions. An ideal design would be a case-control study, in which near-miss cases are compared with non-near-miss caesarean sections for risk factors, surgical findings and interventions. A similar study should also be done in a resource-poor setting, where access to technological and specialist interventions is restricted, and where there are likely to be more maternal deaths.

## Conclusion

Our findings show that maternal near-miss from BDACS is uncommon, but severe enough to necessitate extensive life-saving interventions. These require skilled or specialist health professionals, and a functional obstetric service with access to medication, blood transfusion, emergency transport and intensive care units. An important factor in initiating the sequence of interventions is the realisation by the surgeon that the caesarean section is difficult, so that the progression from uneventful operation to near-miss to death can be arrested.

## Abbreviations

BDACS: Bleeding during and after caesarean section
g: Gram
g/dl: Gram/decilitre
HIV: Human immunodeficiency virus
mL: Millilitre
n: Number
p: Probability
v.: Versus
WHO: World Health Organization
